# A Practical Treatise on the Operation of Surgical and Mechanical Dentistry

**Published:** 1847-10

**Authors:** S. P. Hullihen


### ARTICLE IX.

*A Practical Treatise on the Operation of Surgical and Mechanical Dentistry.* By SAMUEL C. HARBERT, Surgeon Dentist, Philadelphia. BURRIT & JONES, Printers—1847.

THE above is the title of a book—yes—a book of two hundred and six pages; with a most imposing index, and a preface of exceedingly modest dimension—a preface in which the author puts forth the astounding statement, (and by his book, he, of course, offers to prove the fact,) that "Several useful works have been published on the operations of dental surgery, while the mechanical department, *as yet in its infancy*, has received but little attention."

If, then, the works of Drs. Harris, Brown, Maury, Fox, Robinson, and of a host of others heretofore supposed eminent writers on mechanical dentistry, have been only doing up the subject in its baby clothes—what note of praise is long, loud, deep, immense enough to do but common justice to our author, who, of course, presents the subject now full grown, and fully developed:

> "Hail chieftain of the dental art,
> To thee its votaries kneel,
> Their eyes stick out like skillet-legs,
> So thankful do they feel."

But more than this: Our author with that cool, quiet, self-complacency, which a long life of great and varied professional experience, together with a mind of supreme mental endowments can only give, fully conscious that he is treating of a subject "*as yet in its infancy*," one that he has "solitary and alone," reared up to its full meridian, says of its work, "It was designed in the publication of this volume, to fill an existing void in the mechanical part of dental practice, and at the same time to aid the practitioner in dental surgery, *by means of the author's experience and research.*" Mark that: the author's experience and research—how magnanimous, very—how unsophisticated his dignotion.

But this is not all—our author proceeds: "A life of practical experience, exercised with the growing operations of the art, and in the theatre of its most successful experiments, *form the basis of the writer's resources*, from which he has drawn liberally, and unreservedly, withholding nothing he considered important in the prosecution of the dental art," and this, dear reader, will appear, in the sequel, as clear as "mud."

We must not, however, at this place forget, for the benefit of those who may wish to procure instruments, teeth, and a few other things consonant with our author's "experience and research," to mention that these matters have been very thoughtfully provided for by the author, and illustrated in four extensive plates—which plates may be found most cunningly arranged, between the index and preface of the book, instead of being scattered throughout the pages of the work, for the purpose of illustrating, more clearly, important matters in the text; a plan that has been so ignorantly adhered to by most writers who have preceded our author, and as there is no one thing that will exhibit the character of our author's "resources" so graphically as his cuts, we will now notice them in detail:

Plate 1, Fig. 1, represents five excavators, one burr-headed drill, and one common drill, and one socket handle as fitted to the whole lot, all copied if I mistake not, from the celebrated works of Dr. Paré, who flourished during the fifteenth century. Figs. 2, 3, 4, represent tooth-keys in the grammatical comparison of big, bigger, biggest; and copied without the slightest alterations, from a German work published before the time of Paré.

Plate 2, Figs. 1, 2, 4, 5, represent artificial teeth with gums "of brick-dust hue." Fig. 3, represents teeth mounted on plate, from which an important improvement of the author sticks out on one side, almost three-fourths of an inch. Fig. 6, is *not* a representation of the author and his book, but of a bellows, and a fixture, which no doubt would be as useful to the Humane Society for the Restoration of the Drowned, as it will be to the dental profession.

Plate 3, Fig. 1, represents a crooked pair of forceps without any observable peculiarity. Fig. 2, a plaster cast mounted with beeswax, &c.

Plate 4, Fig. 1, represents a punch of doubtful qualifications. Fig. 2, a spiral spring of a very familiar aspect; this constitutes the finale of our author's group of ancient and of homogeneous cuts.

> "Oh! how edifying 'tis to see
> The empty comb of a humble bee."

Part 1st. Chapter 1st. The history and importance of the dental art.

We dislike our author's use of the word *art*, its import is so equivocal, so like trade, it is so like hauling our standard down among the mere mechanical pursuits of life, instead of rearing it aloft side by side with the most useful sciences of the age, and there maintaining it, not only by the correctness of our theory and practice, but by every *word, deed and action.* Our author commences: "The necessity of knowledge in the anatomical structure and organization of the teeth; and the treatment of diseases to which they are subject, are matters of more importance than would be supposed from the practice of *a majority of dental operators*, who content with the merest exhibition of mechanical ingenuity," &c. He affirms that such "operate in all cases alike,"—"to the serious inconvenience and comfort of the patient;" that they destroy many teeth "that might have been, by judicious treatment, preserved for life." He laments that the dental profession is in the hands of such "charlatans and imposters," and that "no penal enactment can reach them." This is all very sensible, and, doubtless, very true. But mark what follows: He says, "These remarks, are not designed to apply to *the mass of dental operators.*" And now, pray, what are we to understand by "the mass of dental operators," if it does not mean "the majority of dental operators." Our author, doubtless, knows—we do not—and so

"We'll pass along to spots more clear."

Our author proceeds, "a ray of hope has dawned upon the dental *art* in this country, by the establishment of collegiate institutions in the cities of Baltimore and *Philadelphia.*" Now, how on earth our author could have made such a mistake as to locate one of the dental colleges in Philadelphia, in the very city in which he resides, instead of Cincinnati, where it is well known that college has been in operation for the last two or three years, can only be imagined by supposing that he has drawn upon his own "resources" for this important historical fact.

Our author proceeds—he next complains that the "science of dentistry has made but a slothful progress compared with other arts," that Hippocrates, "in his writings, treated of the diseases and functions of the teeth," and likewise "Celsus, a Roman physician," and "many other physicians of antiquity;" and here our author all at once fizzles, fizzles, fizzles out, without saying one word on "the importance of the dental art," as we had a right to expect from the heading of this chapter—

> "O, dear! O, dear! what can the matter be,
> O, blood and zounds! what can the matter be,
> And they all cried out, what can the matter be,
> He's bothered from head to the tail."

Thus far have we gone with our author, and thus far have we given a full outline of our author's remarks, sufficient we trust to convey a tolerable correct idea of his "resources" and literary abilities. We now propose to notice, and very briefly, a few passages from the body of his work, with the view of shadowing forth the author's "experience and research" in a professional point of view, and then close with a few general remarks.

Our author, like other writers who have preceded him, treats of his subject under different heads, and these different heads are very like those of other writers, almost as much so as the similarity of the matter, which in most chapters amounts to about the same thing, save now and then, when our author is less full and clear than his cotemporaries.

The following example, although a little more full and original than is common with our author, is very characteristic of him. In treating "of a proper regard to the health and permanency of the teeth," he uses the following language: "Abscesses of a serious nature are frequently produced in the gums by cold striking against the nerve of a decayed tooth. This state of things is not unfrequently characterised by severe general symptoms, such as pain, first centred in the gum, extending itself to the eye, nose and ears; head-ache, restlessness, *catarrh, cough, occasionally diarrhœa and dysentery; dejection and oppression of spirits;* fever, unusual irritability, furred tongue, *spasms, fits, and convulsions;* in females, high fever and delirium."

From this we are to infer that pain and inflammation in the gums—besides a host of other woes—cause males to have

*spasms, fits and convulsions; in females, high fever and delirium.* Thus our author, in the language of Moore,

> "———Wraps nonsense round
> In pomp and darkness, till it seems profound;
> Play on the hopes, the terrors of mankind
> With changeful skill. * * * * * * * *
> While reason, like a grave-faced mummy, stands
> O'erwhelmed with lies and misplaced words."

Our author has doubtless some ambition in the way of being quoted by the temperance orators of our day, which alone can explain the following isolated paragraph respecting a very intemperate woman. He says, "after making way with all her substance, and being without the means of procuring drink, she actually sold all her teeth but two to procure her favorite beverage—gin. The price she received was four pence each." A somewhat similar affair must have happened in the days of Robin Hood, which is duly announced and condemned by his historian in the following lines:

> "To sell her bones was very bad,
> But to buy them was much badder."

We now turn over several chapters to notice our author's opinion of the effects of tobacco upon the teeth. He says, "that it is injurious, may be inferred from two circumstances: first, the action upon the gums, and thence indirectly upon the vital functions of the tooth; and secondly, by absorption of its active properties into the bony substance of the tooth. * * * * * * The teeth not only externally, but upon an anatomical examination, will be found highly impregnated with a *yellow fluid*, evident only in the teeth of persons who use tobacco."

This same *yellow fluid*, which our author describes as being found in the teeth of tobacco-chewers, is likewise referred to by a Mrs. Caudle, of a body, in the following lines, which is the only authority we can find that goes in the least towards supporting the correctness of our author's discovery:

> The quid goes in, when the pipe goes out,
> 'Tis chew, chew, chew;

Now a cloud of smoke pours from his throat,
Then his mouth sends a *yellow stream* afloat,
Sufficient to carry a mill or a boat—
'Tis chew, chew, chew."

A few chapters further on, our author discourses on the "treatment of caries," and of the best material for plugging teeth. He says of gold, "Large cavities are sometimes considered not worth filling with this metal. My own opinion is, *that a tooth worthy to be filled, is worth filling with gold.* When it is past the hope of remedy, it had better be extracted, than to prolong the inconveniences, and evils of it, by useless experiments. This doctrine, "that a tooth worthy to be filled, is worth filling with gold," is by no means peculiar to our author. It long since became a fixed principle of practice, with all well informed *sane* dental surgeons, and one to which they adhere with the most uncompromising fidelity. How is it with our author? A few pages further along, he treats on the "Filling of teeth." Hear him. "Cement, for teeth *presenting a mere shell*, or for roots that a patient may be anxious to retain, will be found highly useful, and not as objectionable as is generally imagined; it can be applied without the preparation that should be exercised previous to filling with other substances, and soon acquires a hardness that will resist any ordinary attrition. I have used it in large quantities, and have never found any bad effects from it; it is also used by many practitioners whose celebrity should entitle their judgment to some respect; by their practice, they must agree with me, in the utility and harmlessness of it."

Thus it will be perceived, that our authcr "halts not between two opinions." He goes for gold plugs, and for nothing else but gold plugs; and then, again, he goes for cement, and for cement as far as the most vehement "amalgamite" could desire. He goes for extracting all teeth "that are not worthy of being filled with gold," and then again he goes for filling with cement, "teeth presenting a mere shell." In short, our author appears to go for anything that may tend towards making up a book. This, the second part of his work goes to show even more plainly than the first; and as we have already quoted from our author quite sufficiently, we trust, to convey a very general impression of his book, we will close our review here, deeply impressed with this conclusion, that our author is very much like unto a small necked bottle, "the less there is in it, the more noise it makes in pouring that little out."

The time has come, when, doubtless, much attention will be directed towards dental literature. Many books will be yearly thrust before the profession, some emanating from the pens of well-educated and well-informed dental surgeons, but many more from vain, impertinent and ignorant dental pretenders. And how are the profession at large to distinguish the works of the worthy from those of the unworthy? How are they to be warned of the imposter and his impositions? There is no way that we know of but one, no plan but to review all works of a dental character, so soon as they fall from the press, and to review them fully, fairly and fearlessly. This course will not only put down effectually and speedily, the frothy puff-books of the pretenders, but it will likewise have the tendency to advance the works of the true dental surgeon, and to bring them to the notice of the whole dental profession, and not only of the dental profession, but of the medical world; and here we would add, that it is the approbation and support of the medical profession that dentists should seek to secure, if they would rise in professional standing; and they can only hope to secure this approbation, by the correctness of their principles and practice.

S. P. HULLIHEN.

